# Comparison of artificial intelligence and rheumatologists in nailfold capillaroscopic evaluation

**DOI:** 10.1590/1806-9282.20251389

**Published:** 2026-05-08

**Authors:** Neşe Çabuk Çelik, Elif Altunel Kılınç

**Affiliations:** 1 Sincan Training and Research Hospital, Department of Rheumatology – Ankara, Türkiye.; 2 Mersin Training and Research Hospital, Department of Rheumatology – Mersin, Türkiye.

**Keywords:** Nailfold capillaroscopy, Artificial intelligence, Diagnostic imaging, Scleroderma, systemic, Deep learning

## Abstract

**OBJECTIVE:**

The aim of this study was to evaluate the diagnostic accuracy of a pretrained Vision Transformer model on nailfold capillaroscopy images, comparing its performance to that of expert rheumatologists.

**METHODS:**

We retrospectively analyzed 104 anonymized images (23 normal, 81 pathological) from publicly available datasets. The pretrained Vision Transformer model was applied without fine-tuning. Two rheumatologists independently assessed the same image set. Accuracy and interrater agreement were calculated.

**RESULTS:**

The AI model produced 0% clinical applicability, failing to generate meaningful classifications. In contrast, the consensus of two rheumatologists achieved the highest diagnostic performance, with 94.2% accuracy and a Cohen’s Kappa of 0.827. Individual rheumatologist evaluations yielded comparatively lower accuracy and agreement.

**CONCLUSION:**

We retrospectively analyzed X anonymized images (XX normal, XX pathological) from publicly available datasets. The pretrained Vision Transformer model was applied without fine-tuning. Two rheumatologists independently assessed the same image set. Accuracy and interrater agreement were calculated.

## INTRODUCTION

Systemic sclerosis (SSc), a rheumatological disease, is a complex autoimmune disorder characterized by vascular abnormalities and fibrosis^1^. Raynaud phenomenon, a common early manifestation of SSc, results from impaired digital perfusion and may also occur as a primary condition or secondary to various underlying diseases^2^.

Nailfold capillaroscopy (NFC) is a widely utilized non-invasive technique for evaluating microvascular abnormalities in patients with systemic sclerosis and other connective tissue diseases. Expert rheumatologists traditionally interpret NFC images to detect abnormal capillary patterns associated with scleroderma-spectrum disorders. However, this manual evaluation is time-consuming and subjective, thus highlighting the need for automated diagnostic solutions^3^.

Artificial intelligence (AI) has recently gained traction in dermatology and nail disease diagnostics^4,5^. Convolutional neural networks (CNNs) have achieved significant success in dermatologic image analysis, including nailfold pathology, enhancing both diagnostic accuracy and efficiency^5,6^. Kassani et al. demonstrated the feasibility of AI-based capillaroscopy in juvenile dermatomyositis, showcasing the potential of AI applications in rheumatology^7^. However, the clinical translation of general-purpose models to medical domains remains challenged by limited adaptability and a lack of domain-specific datasets^8,9^.

Vision Transformer (ViT) models have emerged as competitive alternatives to CNNs in image classification by leveraging self-attention mechanisms. While promising in general tasks, their application to specialized medical imaging, such as capillaroscopy, remains underexplored^5,9^.

In this study, we assessed the diagnostic utility of a pretrained ViT model when applied to nailfold capillaroscopy images without any additional domain-specific training. We compared its output with expert rheumatologist evaluations and literature-based criteria to assess the feasibility of using such general-purpose AI tools in clinical capillaroscopic assessment.

Although AI has previously been applied to nailfold capillaroscopy in specific contexts such as juvenile dermatomyositis^7^, to our knowledge, no prior study has directly compared the diagnostic performance of an AI model against expert rheumatologists. This study represents the first attempt to evaluate the clinical applicability of a general-purpose ViT model in capillaroscopic analysis using a comparative design.

## METHODS

### Study design and image collection

This retrospective validation study was conducted using publicly available and anonymized nailfold capillaroscopy images. All images were collected from open-access scientific publications, Creative Commons–licensed datasets, and Wikimedia Commons. A total of 104 images were selected, representing both normal and abnormal nailfold capillaroscopic patterns, including features consistent with scleroderma-spectrum disorders. Images were selected based on the following criteria: sufficient resolution to visualize capillary morphology (typically ≥400× magnification), clarity without significant motion blur or artifact, and a balanced distribution of normal and abnormal findings. Preference was given to images with published expert interpretation or source-provided diagnostic labels. As no patient-identifiable data were used, ethical approval was not required.

### Artificial intelligence assessment

For the AI-based evaluation, we used a pretrained ViT model (“google/vit-base-patch16-224”), accessed through the Hugging Face platform. This model was originally trained on the ImageNet dataset for general-purpose object classification and was used without any domain-specific fine-tuning or additional training.

Each image was uploaded individually, and the model’s top-1 predicted class and associated confidence score were recorded. Since the model was not trained on medical data, the predicted categories were not expected to reflect clinical labels.

Clinical applicability was defined as the percentage of AI-generated predictions that were medically interpretable and relevant to capillaroscopic diagnosis. Irrelevant predictions (e.g., “accordion,” “nematode”) were classified as non-applicable. All AI evaluations were conducted using Python (v3.13) and the Hugging Face transformers pipeline. Output data were compiled and analyzed in Microsoft Excel.

### Rheumatologist assessment

Notably, two independent board-certified rheumatologists reviewed all 104 images. Each evaluator classified the images as either normal or abnormal (presence of scleroderma-spectrum changes). In cases of disagreement, a consensus decision was reached through joint review. If disagreement persisted, the classification from the original literature source was adopted as the final label. In the individual performance analysis, the first rheumatologist achieved an overall accuracy of 86.5%, with high sensitivity (93.8%) and moderate specificity (60.9%), whereas the second rheumatologist showed lower accuracy (79.8%), despite a high sensitivity (97.5%), but markedly low specificity (17.4%). This disparity suggests that the second rheumatologist may have favored sensitivity over specificity, potentially overcalling abnormalities. In contrast, the first rheumatologist demonstrated a more balanced diagnostic approach. These findings highlight inter-rater variability in capillaroscopic interpretation and underscore the value of consensus evaluations, which showed superior diagnostic performance across all metrics.

### Statistical analysis

Statistical analyses were performed using Python (v3.13) and the scikit-learn library. The literature-based diagnosis was considered the gold standard.

Diagnostic performance of each rheumatologist and their consensus decision was evaluated using:

- Accuracy (proportion of correct classifications),
- Sensitivity (true positive rate),
- Specificity (true negative rate),
- Cohen’s Kappa coefficient (for interobserver agreement beyond chance).

Because the ViT model could not produce clinically meaningful outputs, traditional diagnostic metrics (e.g., accuracy, sensitivity) were not calculated for the AI. Instead, clinical applicability was reported as a descriptive metric.

**Figure 1:**
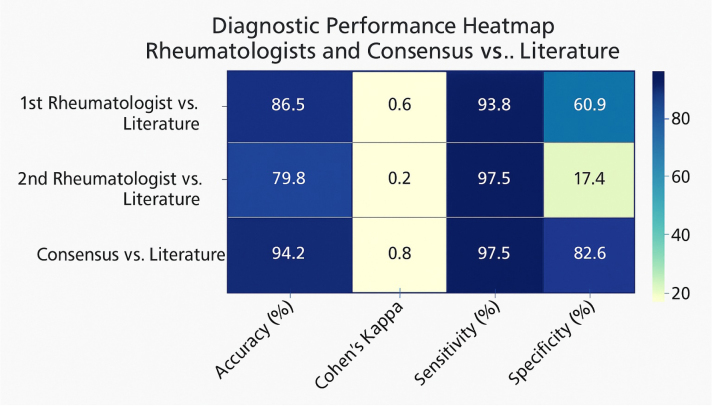
Diagnostic performance heatmap rheumatologists and consensus vs. literature.

Results are summarized as descriptive statistics and visualized using heatmaps and bar charts (see Figure 1).

### Ethical considerations

All images used in this study were anonymized and sourced from publicly available materials. As no patient-identifiable information was used, institutional ethical approval was not necessary.

## DISCUSSION

This study evaluated the clinical applicability of a pretrained ViT model on nailfold capillaroscopy images without any domain-specific training. To our knowledge, this is the first study to apply a general-purpose ViT model to this imaging modality and directly compare its performance with expert rheumatologist evaluations.

**Figure 2:**
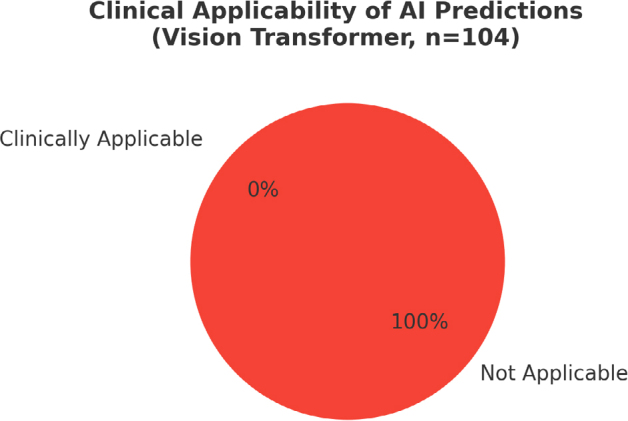
The clinical applicability of artificial intelligence predictions.

The ViT model failed to produce any clinically meaningful predictions, with 0% clinical applicability (Figure 2). In contrast, the consensus assessment by two rheumatologists achieved high diagnostic accuracy (94.2%) and substantial interobserver agreement (Cohen’s Kappa: 0.827). These findings emphasize the inherent limitations of directly deploying general-purpose AI systems in specialized medical imaging tasks.

Earlier studies have demonstrated the success of CNNbased models in dermatology and nailfold pathology, reporting significant improvements in diagnostic performance^5,6^. For example, Kassani et al. developed an AI-based approach for capillaroscopy in juvenile dermatomyositis with promising results^7^. However, these approaches either utilized domain-specific datasets or focused on relatively narrow classification tasks. In contrast, the ViT model in our study was neither fine-tuned nor trained with medical images, which likely explains its poor performance.

The irrelevant AI outputs (e.g., “accordion,” “nematode”) illustrate the mismatch between general-purpose training data and medical imaging requirements, a phenomenon also observed in large language models used for rheumatologic guidance^10,11,12^. Our findings extend these concerns to vision-based AI, underscoring the non-transferability of general-purpose architectures to critical clinical domains without adaptation.

### Clinical implications

From a clinical standpoint, the model’s 0% applicability rate is a red flag for patient safety. If such models were mistakenly used in real-world settings without validation, they could lead to misdiagnoses, incorrect treatment decisions, and delayed care. Physicians and healthcare institutions must be cautioned against the premature adoption of unvalidated AI tools, especially in diagnostic pathways involving high-stakes outcomes.

### Recommendations for future research

To bridge this performance gap, future research must prioritize:

- Transfer learning with capillaroscopy-specific image features
- Construction of large-scale, annotated datasets curated by expert clinicians
- Use of explainable AI (XAI) tools, such as heatmaps or attention maps, to ensure model interpretability
- Hybrid architectures combining CNN and transformer models, leveraging the strengths of both
- Establishment of standardized benchmarks, including comparison with multiple human raters as performed in this study

Such efforts would not only improve model performance but also enhance clinician trust in AI-assisted diagnostics.

## CONCLUSION

**Figure 3:**
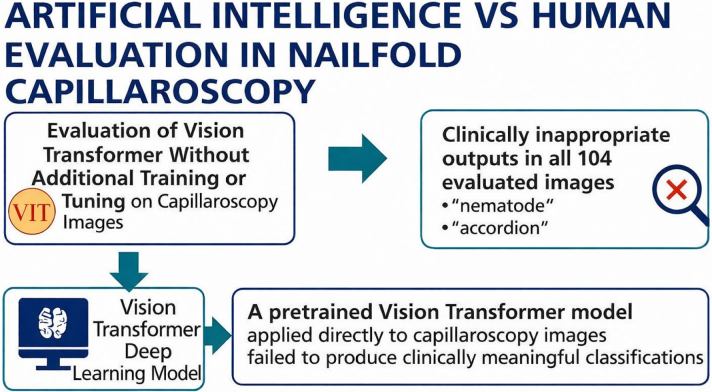
Graphical abstract showing the comparison between a pre-trained Vision Transformer model and expert rheumatologists in nailfold capillaroscopic interpretation. The artificial intelligence model demonstrated 0% clinical applicability, while rheumatologist consensus achieved high diagnostic accuracy.

In summary, general-purpose pretrained ViT models are not suitable for capillaroscopic image interpretation without domain adaptation. Until robust, validated, and explainable AI models are developed through collaborative clinical–AI partnerships, expert rheumatologist assessment must remain the gold standard to ensure diagnostic accuracy and protect patient safety (Figure 3).

## Data Availability

The datasets generated and/or analyzed during the current study are available from the corresponding author upon reasonable request.
