# ERRATUM: Comparison of artificial intelligence and rheumatologists in nailfold capillaroscopic evaluation

**DOI:** 10.1590/1806-9282.20251389-ER

**Published:** 2026-07-31

In the manuscript: Çelik NÇ, Kılınç EA. Comparison of artificial intelligence and rheumatologists in nailfold capillaroscopic evaluation. Rev Assoc Med Bras. 2026;72(2):e20251389. https://doi.org/10.1590/1806-9282.20251389, on page 1, SUMMARY:

**Where it reads:**

CONCLUSION: We retrospectively analyzed X anonymized images (XX normal, XX pathological) from publicly available datasets.

**It should read:**

CONCLUSION: We retrospectively analyzed 104 anonymized images (23 normal, 81 pathological) from publicly available datasets.

